# Development of the competency-based medical curriculum for the new Augsburg University Medical School

**DOI:** 10.3205/zma001098

**Published:** 2017-05-15

**Authors:** Anja Härtl, Pascal Berberat, Martin R. Fischer, Helmuth Forst, Stefanie Grützner, Thomas Händl, Felix Joachimski, Renate Linné, Bruno Märkl, Markus Naumann, Reinhard Putz, Werner Schneider, Claus Schöler, Markus Wehler, Reinhard Hoffmann

**Affiliations:** 1 Klinikum der Ludwig-Maximilians-Universität München, Institut für Didaktik und Ausbildungsforschung in der Medizin, München, Deutschland; 2 Technische Universität München, Medizin-Didaktisches Centrum für Ausbildungsforschung und Lehre TUM MeDiCal, München, Deutschland; 3 Klinikum Augsburg, Klinik für Anästhesiologie und operative Intensivmedizin, Augsburg, Deutschland; 4 Klinikum Augsburg, Institut für Transfusionsmedizin und Hämostaseologie, Augsburg, Deutschland; 5 Klinikum Augsburg, IV. Medizinische Klinik, Augsburg, Deutschland; 6 Klinikum Augsburg, Klinik für Neurologie und klinische Neurophysiologie, Augsburg, Deutschland; 7 Klinikum Augsburg, Stabsstelle "Aufbau eines Universitätsklinikums", Augsburg, Deutschland; 8 Klinikum Augsburg, Institut für Pathologie, Augsburg, Deutschland; 9 Ludwig-Maximilians-Universität München, Lehrstuhl Anatomie I - vegetative Anatomie, München, Deutschland; 10 Universität Augsburg, Philosophisch-Sozialwissenschaftliche Fakultät, Lehrstuhl für Soziologie mit Berücksichtigung der Sozialkunde, Augsburg, Deutschland; 11 Klinikum Augsburg, Klinik für Allgemein-, Viszeral- und Transplantationschirurgie, Augsburg, Deutschland; 12 Klinikum Augsburg, Institut für Labormedizin und Mikrobiologie, Augsburg, Deutschland

**Keywords:** Curriculum planning, establishing a university faculty, spiral curriculum, hybrid curriculum

## Abstract

**Aim:** With the resolution from April 28, 2014, the Bavarian state government in Germany decided to found a new medical school at Augsburg University, thereby requiring the development of a competency-based medical curriculum.

**Methods: **Two interdisciplinary groups developed a spiral curriculum (following Harden) employing the model of Thumser-Dauth & Öchsner. The curriculum focuses on specifically defined competencies: medical expertise, independent scientific reasoning, argumentation and scholarship, as well as communication skills.

**Results: **The spiral curriculum was developed as a hybrid curriculum. Its modular structure incorporates the mandatory subjects required by the German regulations for medical licensure (Approbationsordnung) into organ- and system-centered blocks which are integrated both horizontally and vertically. Basic preclinical sciences are covered in the blocks “Movement,” “Balance” and “Contact.” The clinical sciences are organized according to six pillars (conservative medicine, surgical medicine, men’s-women’s-children’s medicine, the senses, the nervous system and the mind, and general medicine) which students revisit three times each over the course of the program. A longitudinal clinical course incorporates interdisciplinary education. A particular focus is on scientific education encompassing a longitudinal course in the sciences (including interdisciplinary classes with other university departments), block practicums, and two scientific projects.

**Conclusion:** It is not only the degree of integration und intensity of the Augsburg University undergraduate medical degree program, but also its targeted advancement of academic, social and communication skills that have not yet been realized to such an extent elsewhere in Germany. On July 8, 2016, the German Council of Science and Humanities unanimously gave this concept a positive evaluation. Future research will examine and evaluate the Augsburg medical curriculum and the impact of the new medical school on the hospital and university in Augsburg.

## Zusammenfassung

**Zielsetzung:** Im Kabinett wurde am 28.4.2014 vom Freistaat Bayern die Gründung einer medizinischen Fakultät an der Universität Augsburg und die Umwandlung des kommunalen Klinikums Augsburg in ein staatliches Universitätsklinikum beschlossen. Hierfür sollte ein kompetenzorientiertes Modellcurriculum entwickelt werden.

**Methodik:** In zwei interdisziplinären Arbeitsgruppen wurde gemäß dem Modell von Thumser-Dauth & Öchsner ein Spiralcurriculum (nach R. Harden) entwickelt, das sich an definierten Kompetenzbereichen orientiert: medizinische Fachkompetenz, selbständiges wissenschaftliches Denken, Argumentieren und Arbeiten sowie soziale und kommunikative Kompetenzen.

**Ergebnisse: **Das Spiralcurriculum wurde als Hybridcurriculum angelegt. Der modulare Aufbau berücksichtigt den Fächerkanon der Approbationsordnung durch organ- und systemorientierte Blöcke, die horizontal und vertikal integriert gestaltet werden. Die Grundlagen sind in den Blöcken Bewegung, Gleichgewicht und Kontakt realisiert, während die klinische Medizin in 6 Säulen abgebildet ist, die jeweils dreimal durchlaufen werden (konservative Medizin, operative Medizin, Kind-Frau-Mann-Medizin, Sinnesmedizin, Medizin des Nervensystems und der Psyche, Allgemeine Medizin). Ergänzend wird ein klinischer Longitudinalkurs mit übergeordneten Inhalten realisiert. Besonderer Schwerpunkt liegt auf der wissenschaftlichen Ausbildung. Diese wird durch einen wissenschaftlichen Longitudinalkurs, zwei wissenschaftliche Blockpraktika und zwei Projektarbeiten realisiert. Hieran beteiligen sich auch andere, nicht-medizinische Fakultäten der Universität Augsburg.

**Schlussfolgerung:** Insbesondere die Integration und Intensität der wissenschaftlichen Ausbildung, aber auch die gezielte Förderung wissenschaftlicher, sozialer und kommunikativer Kompetenzen werden im Augsburger Curriculum in bisher nicht beschriebener Weise realisiert. Das Konzept wurde am 8. Juli 2016 vom Wissenschaftsrat einstimmig positiv begutachtet. Neben der wissenschaftlichen Begleitung des Augsburger Modellcurriculums werden auch die Auswirkungen der Fakultätsgründung auf Klinikum und Universität Gegenstand künftiger Forschung sein.

## 1. Introduction

### 1.1. Medical school admission in Germany

There is consensus among several professional organizations that there are not enough spaces to study medicine at German universities. The current shortage of young doctors in hospitals and private practices will become more acute over the next 10 years, especially as many physicians retire [1]. Sufficient numbers of applicants to medical school do exist, but universities lack the capacity to admit and educate them. At present only around 12,600 spaces are available each year for over 50,000 applicants, meaning that even those with excellent secondary school records are not guaranteed admission to a medical degree program [1]. The creation of new medical schools is viewed as one possible strategy to counter this inability to admit more students and to combat the shortage of medical doctors [2], [3]. This is being done partly on the basis of European law: medical degree programs are being created at large German hospitals in collaboration with medical schools in other European countries. The quality of these programs is criticized by the German Council of Science and Humanities primarily for their lack of an academic focus on science [4]. In the Free State of Bavaria the number of spaces to study medicine is marginally above the national German average in terms of the total population (109.2 spaces per 100,000 inhabitants in comparison to 108.2 spaces per 100,000 inhabitants nationwide), but far below other German states, such as Mecklenburg-Western Pomerania (205.3 spaces per 100,000 inhabitants) [5].

The Bavarian state’s intention to promote undergraduate medical education at state schools by founding a sixth medical school at Augsburg University is undoubtedly a response to a clear need.

#### 1.2. Augsburg University

Augsburg University was founded in 1970 as an interdisciplinary university. Approximately 20,000 students are presently enrolled at the university’s seven schools. With 1,700 beds, Augsburg’s hospital is one of the largest in Bavaria and the only tertiary care hospital in the region of Swabia that is home to a population of approximately two million. The hospital, which opened its doors in 1982, was conceived from the start as a future university teaching hospital. However, the first attempt in the 1980s to establish a center for medical teaching and research at Augsburg University was unsuccessful [6]. With the decision of the Bavarian government on July 28, 2014, the intent to establish a medical school at Augsburg University, consisting of a university hospital and a department of medicine, was revived. On June 1, 2015, the Free State of Bavaria requested the German Council of Science and Humanities to evaluate a general concept for a medical school in Augsburg and to share its opinion [5]. A positive evaluation of the German Council was confirmed unanimously on July 8, 2016 [7].

#### 1.3. Reasons behind founding the new medical school in Augsburg

With the founding of a medical school in Augsburg, the capacity to admit medical students in Bavaria will be increased by around 14%; in the final stage of expansion this will mean 252 additional spaces per year. In addition, the newly created medical school enables implementation of an innovative curriculum following the recommendations of the German Council of Science and Humanities regarding the development of medical study in Germany with the long-term objective of qualitatively improving medical care in the Augsburg region by innovatively educating a new generation of physicians [5].

#### 1.4. Aims of the curriculum development

Employability is considered a core objective of the new curriculum under development. This term is defined by the Bologna Process as *“a set of achievements – skills, understandings, and personal attributes – that makes graduates more likely to gain employment and be successful in their chosen occupations, which benefits themselves, the workforce, the community and the economy”* [8].

This is defined specifically for medicine in the German National Competency-based Catalogue of Learning Objectives for Undergraduate Medical Education (NKLM) as the ability to independently practice medicine and pursue advanced post-licensure medical training and education [9].

In addition to employability as a medical doctor, the scholarly development and personal growth of the students are to be fostered and encouraged in a targeted manner by strengthening their academic, social and communicative skills.

Such growth and development are to be enabled by a competency-based curriculum that is horizontally and vertically integrated. This is to be realized by teaching interdisciplinary, organ-centered and system-centered courses combining preclinical, theoretical and clinical subjects. The biopsychosocial model was selected to provide the conceptual framework for the integrated curriculum [10].

The development of the competency-based medical curriculum for the Augsburg medical school and the basics of the curriculum itself are described in the following section. What is presented is an initial report on the early developmental stage and is meant to serve as a guide for the rare process of establishing a new medical school at a state university. Furthermore, a critical and informed examination of the Augsburg curriculum is something to be encouraged. 

## 2. Method

After the decision was made to have the German Council of Science and Humanities establish a medical school in Augsburg, the Bavarian State Ministry for Education and Culture, Science and the Arts (StMBW) appointed a working group on academic study at Augsburg University. This steering committee, comprised of members of the university, the hospital, representatives from the Ministry and external experts, was commissioned with designing and developing an academic concept, including a curriculum for a degree program and integrating it into the overall concept for the new medical school. Three members of this working group (M.R.F. and R.P. [LMU] and P.B. [TUM]), professors from neighboring medical schools serving as experts on research in medical education, generated the basic structure of the degree program which was then further refined as described in the following section.

According to Schaper et al (2014), when designing new degree programs it is important to involve the stakeholders from the start in any development processes regarding the academic program and to actively include them in any changes. For this reason, it is good practice not only to appropriately structure the development process in terms of design, but also to set this up as an organizational development process with the associated communicative and participatory elements [11]. For this reason, a modified form of the model proposed by Thumser-Dauth & Öchsner (2008), with its seven practical procedural steps (see sections 2.1. to 2.7) regarding competency-based curricular design [12], was followed when drafting the first version of the Augsburg curriculum.

### 2.1. Needs assessment with regard for the existing situation and statutory requirements

The intention was to design a curriculum not only to ensure practical clinical education and learning, but also to strongly encourage and foster the academic development and personal growth of the students. It was assumed that special teaching and learning formats would be necessary to achieve this, ones that can be realized only to a limited extent in a conventional degree program. This was the basis for the decision to develop a model degree program.

#### 2.2. Creation of expert steering committees

A second working group was founded within the initial working group appointed by the Bavarian Ministry (StMBW) as an operative expert group to design the Augsburg curriculum (working group on hospital teaching). The intention was to form a mixed group in which experts with different backgrounds and expertise (structural, organizational, clinical, higher education, and medical education) and from different levels of the hierarchy could come together.

Participation was voluntary and open to all medical employees at the Augsburg Hospital. A chief physician familiar with the structural and organizational aspects of the hospital served as committee chairman. In addition, eight hospital physicians (4 chief physicians, 3 senior physicians, 1 assistant physician) actively participated in the working group. Furthermore, it was possible to recruit the Augsburg University’s vice-president for academic affairs, continuing education and equal opportunity as an expert on the university’s structure and organization. Also participating in the curriculum design were three other professors (M.R.F. and R.P. [LMU], and P.B. [TUM]) from neighboring medical schools who served as experts on research in medical education and a university employee specialized in medical education. Within this second working group focused on hospital teaching, the goals and timetables for the planning processes were determined, work packages were assigned, and decisions about the development process were made.

#### 2.3. Inclusion of stakeholders

By combining the two working groups focused on Augsburg University and the Augsburg Hospital, it was ensured that, although the pertinent university structures did not yet exist, all process-relevant institutions were included in developing the curriculum: representatives of the StMBW, Augsburg University, external experts, and physicians practicing at the Augsburg Hospital (most with prior experience in higher education). 

The working group housed at the hospital generated the basic content for development and made regular reports to the working group at the university which, in turn, after holding discussions formally passed the related resolutions. Other institutions were included when necessary.

#### 2.4. Definition of competencies to identify the requisite competency areas

Describing competencies is associated with several problems including the ambiguity of the very word itself [13]. The term “competency” is defined and discussed differently depending on the context. Based on different definitions and descriptions, Schaper has formulated central characteristics of this concept [14]:

- Competency is defined as the ability to act appropriately, responsibly and successfully in specific settings marked by a high degree of complexity, novelty or uncertainty that demand high quality solutions.
- Abilities to act in this manner draw on bundles of complex knowledge, skills, motivational focus, values and attitudes in relation to these specific settings.

In addition, Schaper [14] describes characteristics of academic and professional competencies that cover scholarly work, problem solving and decision making, as well as reflection and communication. With this understanding of competency and based on both the recommendations of the German Council of Science and Humanities [15] and the NKLM [16], the competency areas outlined in section 3.1 were defined.

#### 2.5. Design of a curriculum in alignment with the defined competency areas

Competency-based medical education has been defined by Frank et al. as:

*“Competency-based education (CBE) is an approach to preparing physicians for practice that is fundamentally oriented to graduate outcome abilities and organized around competencies derived from an analysis of societal and patient needs. It deemphasizes time-based training and promises greater accountability, flexibility, and learner-centredness”* [17].

Based on this definition and taking the extenuating logistical situation into consideration, the basic elements of the teaching and learning formats were developed in accordance with Harden’s SPICES model [18]. While doing this, special attention was given to representing the academic and professional competencies in the form of a spiral curriculum [18].

#### 2.6. Focus of the curriculum on the defined competency profile

Detailed development of the curriculum will take place over the next years as part of establishing a medical school at Augsburg University. As this is accomplished, the curricular content, learning objectives, teaching and learning formats, as well as assessment formats, will be coordinated with each other and routinely evaluated in terms of constructive alignment [19].

#### 2.7. Verifying the achievement of goals at the student level and program evaluation

Verification that objectives are being met at the student and program levels can, of course, only be undertaken after the program has been up and running for awhile, meaning after the first student cohorts have graduated. A first draft of a quality assurance concept has been generated for this purpose. This aspect will be developed in parallel to the curriculum.

## 3. Results

### 3.1. Definition of the desirable competency areas for the program graduates

The aim of the degree program is to enable the practice of medicine in the clinical setting and in academic research. The following competency areas were identified building off of the understanding of competency stated in section 2.4:

- Medical expertise (knowledge, skills and abilities)
- Independent scientific reasoning, argumentation and scholarship
- Social and communication skills

As a result, the new degree program will have a primary focus on imparting both the ability to act responsibly in a solution-oriented manner in complex medical situations and the ability to engage in academic scholarship and apply scientific concepts to complex topics [14]. Social and communication skills will not just encompass communication with colleagues, patients or their relatives, but also self-regulation and reflection on the physician’s own actions and decisions, as well as the communication of scientific knowledge, concepts and methods [14].

#### 3.2. Design of the curriculum in alignment with the defined competencies

##### 3.2.1. Basic concept

**Figure 1:**
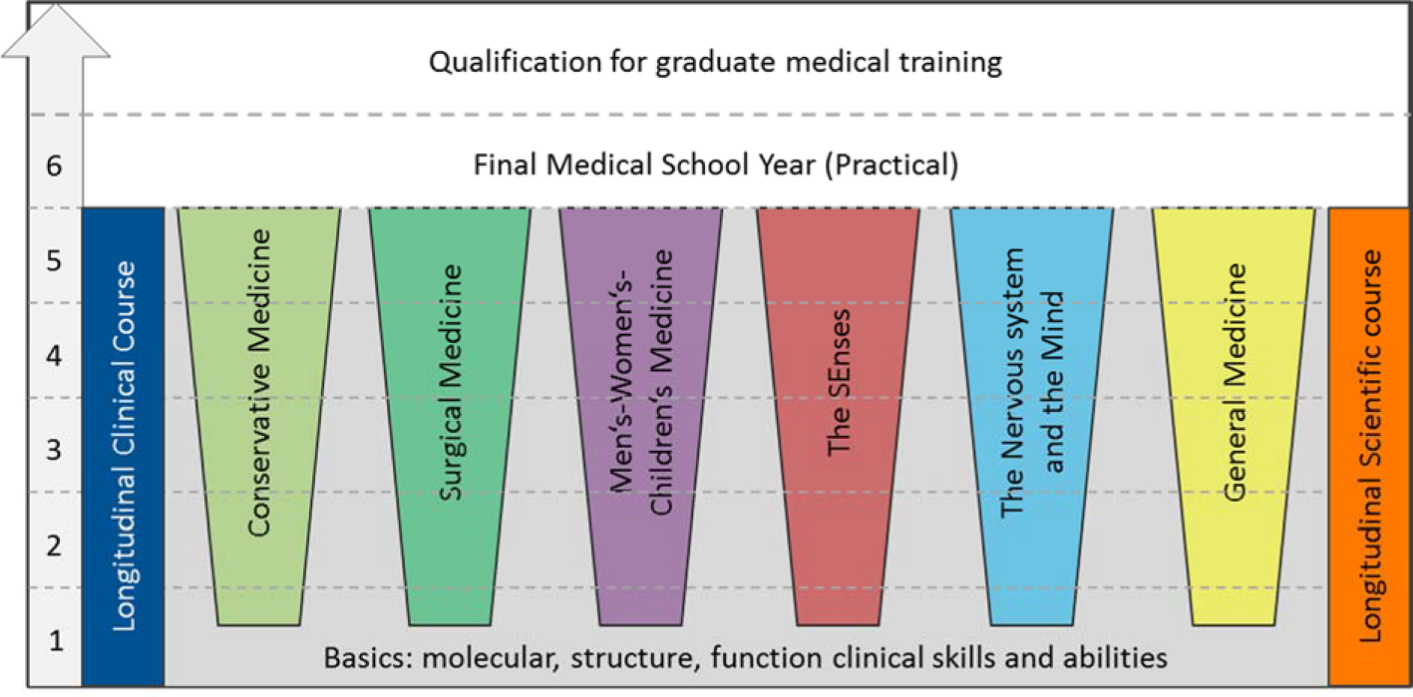
Conceptual framework for the Augsburg competency-based medical curriculum. The traditional preclinical and clinical content is taught in an integrated manner in the first through fifth years of study, whereby the preclinical content decreases as study advances, and the clinical content increases.

Based on the competency areas described here, a rough concept was initially laid out for the Augsburg curriculum (see figure 1 (Fig. 1)). A spiral curriculum was developed [18] in which the separate topics are taught and learned over the entire course of the program with an increasing degree of complexity. Attention was given here to ensuring that traditional preclinical and clinical content can be taught, and thus learned, in an integrated manner during all of the academic years up through the fifth practical year. In addition, two longitudinal courses are planned which continue from the first semester to the end of the fifth year. The academic longitudinal course focuses on enabling scientific reasoning, academic argumentation and scholarship. The clinical longitudinal course is meant not only to promote employability through the application of medical expertise, but also to encourage the acquisition and development of interpersonal and communication skills.

**Figure 2:**
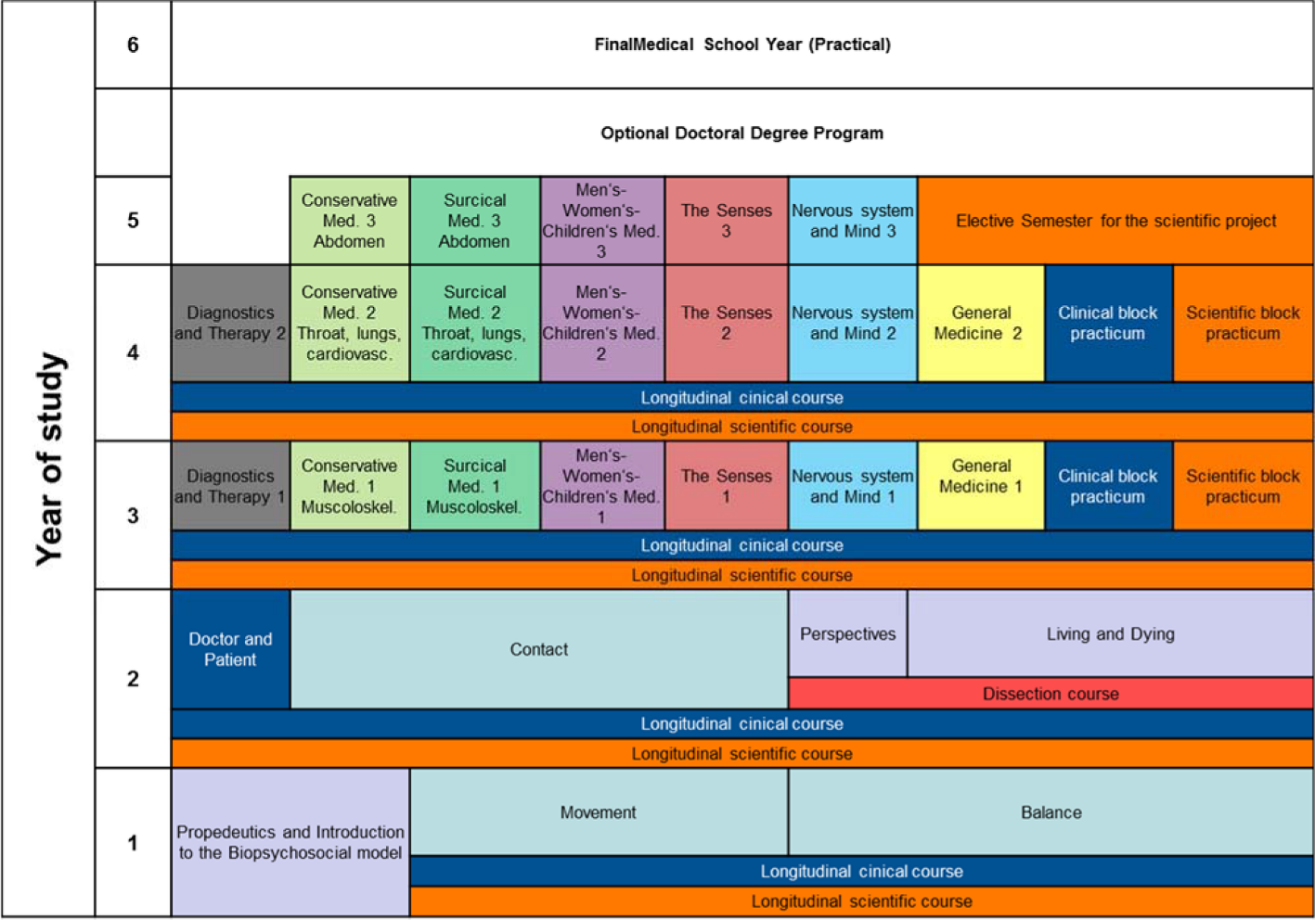
Example illustration of the organ-centered and system-centered blocks of the Augsburg curriculum.

To design the individual areas, blocks were put together in which the preclinical, theoretical clinical, and practical clinical disciplines work together thus guaranteeing horizontal integration in terms of interdisciplinary study. The blocks are organized according to organ-centered and system-centered content. An illustration is given in figure 2 (Fig. 2).

##### 3.2.2. First and second years of study

The first year of study begins with an introductory block on the principles of cell biology, physics, chemistry and biology. The aim is to homogenize students’ knowledge, which is not expected to be uniform at this stage. The guiding principle of the degree program, the *biopsychosocial model* [10], is also introduced. The conventional preclinical content in the subjects of anatomy, physiology, and biochemistry are imparted in three topical blocks during the first two academic years: *Movement *(musculoskeletal system, respiration, cardiovascular system), *Balance *(function of the internal organs, homeostasis) and *Contact* (sensory organs, nervous system, psychology, sociology, immunology, microbiology). Delineations between the three core topical blocks are maintained. Clinical relevance will be consistently underscored by weekly patient consultations and by the accompanying presence of the clinical and theoretical clinical subjects. Practical skills, specifically leading symptom anamnesis, examination, diagnostic investigations, and the principles of pathophysiology, are integrated into these areas. Content dealing with anatomy is learned using living persons and models as preparation for the dissection course at the end of the second year of study. As a result, students will already have a wide knowledge of anatomy once they begin the dissection course; repetition of this knowledge using a cadaver is meant to enhance, consolidate, and integrate this anatomical knowledge (e.g. topographic anatomy). Moreover, at the end of the second year the closely linked blocks *Perspectives* (patient perspectives, system perspectives, observations at healthcare institutions besides the hospital) and *Living and Dying* (progression, prevention, major diseases of affluence, geriatrics, intensive medicine, death and dying) enable students to acquire an integrative perspective on the material learned thus far.

##### 3.2.3. Clinical study phase

The third through fifth years of study are predominantly organized into thematic and system-centered blocks. At the beginning of both the third and fourth years there is a block on Diagnostics and Therapy introducing diagnostic investigations and lab-based diagnostics, general pathology, and pharmacotherapy. This serves as preparation for the subsequent six clinical blocks. The required subjects defined by the medical licensure regulations (Approbationsordnung) are represented in six blocks in the Augsburg curriculum: Conservative Medicine, Surgical Medicine, Men’s-Women’s-Children’s Medicine, The Senses, The Nervous system and the Mind, and General Medicine. These blocks are, in turn, made up of sub-blocks taught in the third, fourth and fifth years of study so that students attend three separate blocks over three years on each one of the six topics above (see figure 2 (Fig. 2)). By doing this, it is possible to realize a spiral curriculum not only to consolidate and deepen knowledge and skills through repetition, but also to adjust the complexity of the content for the individual topic areas according to student knowledge. The sequence of the blocks covering conservative medicine and surgical medicine, for instance, enable the topics to be viewed in more depth from different perspectives (see figure 2 (Fig. 2)). The focus of the block on general medicine is on the interdisciplinary view regarding clinical subject matter, for example, in the context of emergency medicine or geriatrics.

##### 3.2.4. Longitudinal courses and block practicums

The curriculum is supplemented by two longitudinal courses. Starting in the first year of study, the longitudinal course in clinical medicine teaches clinical subject matter from different perspectives that go beyond simply caring for patients in a hospital setting. Alongside anamnesis, physical examination and holding medical consultations, overarching subject areas are integrated into the more advanced phase of study, such as the history of medicine, theory and ethics in medicine, medicine and society (healthcare system, management, economics), communication and cooperation, as well as intra- and interprofessional collaboration.

The longitudinal course in science begins in the first years of study and covers the basic principles of scientific work. This includes reading and critically evaluating scientific literature, knowledge of medical research methodologies and their scientific and sociological contexts. Central to the methodology of the first two academic years is the planning and realization of a small-group, scientific research project. In the more advanced years, knowledge of research basics is expanded to reflect more specialized knowledge regarding statistics, qualitative methods, etc. In terms of electives, courses are offered in conjunction with the other academic departments at Augsburg University.

The two longitudinal courses are complemented by related block practicums. The clinical block practicums may be served at hospitals, private practices or other healthcare institutions (e.g. statutory health insurers, government health authorities). The scientific block practicums may be served at any of the institutions of the Augsburg University and even at other research institutions; they may also function as preparation for the fifth-year academic project which is mandatory for all students. In addition, a doctoral program is offered to which students may apply.

##### 3.2.5. Teaching formats

In regard to teaching formats, the Augsburg curriculum is a hybrid one. Starting in the first year of study, both problem-based learning (small-group tutorials) and interactive seminars and practicums (group work and lab practicums) will be established as forms and methods of teaching. Lectures are interactive (inverted classroom) and offered only as an accompaniment to the core curriculum.

The blocks themselves will be designed according to the principle of constructive alignment [19] once the corresponding catalogues of learning objectives have been formulated. This applies to the methods and formats for teaching and assessment.

Instruction in each individual block will be based on the SPICES model put forth by Harden [18]. A focus on students (S: student-centered) and the demand for less information gathering and more problem-based teaching (P) and integration (I) will be reflected in the design of the relevant integrated blocks and subject areas and the selection of teaching formats and methods. Since instruction will take place not just in the hospital setting, it will be possible to pursue a community-based approach (C), particularly in the longitudinal courses and block practicums. In addition, all students should be given the opportunity to choose electives (E). This will be possible in that practicums and courses may be freely chosen within the scope of the longitudinal courses. The systematic approach (S) will be promoted by defining clear teaching and learning objectives.

##### 3.2.6. Assessment formats

Both summative and formative assessment formats will be used. After completing the second year of study, a summative equivalency test corresponding to the first section of the state medical exam will be administered. Furthermore, an assessment is planned after completion of the final year to demonstrate learning outcomes for the fifth and final year focused on practical studies. In addition to established assessment formats such as multiple-choice exams and Objective Structured Clinical Examinations (OSCE) [20], other assessment formats will also be used, for instance, the Objective Structured Long Examination Record (OSLER) [21] and Mini-Clinical Examination (Mini-CEX) [22]. The scores on the formative and summative assessments should be compiled for programmatic assessment [23] using e-portfolios [24], [25]. 

## 4. Discussion and outlook

By following the process described above and involving representatives from all stakeholders, it has been possible to develop a conceptual framework for a new competency-based curriculum while taking current recommendations into consideration. Moreover, the following competency areas are represented in the curriculum:

- **Medical expertise** is imparted in the modularly structured part of the curriculum with the preclinical blocks (Movement, Balance, and Contact), the dissection course as well as through the six pillars of clinical medicine, supplemented by the blocks on diagnostics and therapy and the clinical block practicums.
- **Independent scientific reasoning, argumentation and scholarship **are fostered by the three pillars of the scientific curriculum—the longitudinal course in science, the scientific block practicums and the academic projects—with the possibility of subsequently entering the doctoral program.
- **Social and communication skills** are promoted by integrating these competency areas into the interdisciplinary blocks during the first two years of study (introductory courses and the blocks Doctor and Patient, Perspectives, and Living and Dying), the longitudinal clinical course and the clinical block practicums.

Specifically the level integration and intensity of the university teaching, in collaboration with all of the academic departments of Augsburg University, have been realized in the design of this medical curriculum to an extent not yet seen elsewhere in Germany. Central characteristics of the Augsburg curriculum – which can be implemented as a model degree program – include an emphasis on problem-based, seminar-based, small-group instruction, a delineation between subjects despite horizontal and vertical integration into the curriculum, while turning away, for the most part, from large-group lectures.

Since the medical school has not yet been formally founded, this conceptual groundwork needed to have been done by the affected staff at the Augsburg Hospital, the neighboring medical schools in Munich and the Augsburg University in addition to their regular duties. This cooperative endeavor has been reported as constructive and productive by all those involved. This kind of interdisciplinary cooperation requires very strict organization, particularly among practicing physicians with professional duties in intensive care units, operating rooms and as emergency medical personnel. In general, successful working group activities relied on clear coordination and communication of tasks and pressing ahead with the process even when not all working group members were able to be present at each meeting.

When planning the first phase of establishing any medical school there is no choice but to leave many questions still open. This affects not only aspects related to content (how should a learning objectives catalogue based on NKLM be implemented?; How will the individual blocks be designed in terms of teaching and content?) but also the logistical aspects of the new medical school. Continuation of the conceptual planning must now be preceded by appropriate human resource planning and the creation of the pertinent academic, administrative and institutional structures. Among these are a Dean of Studies, a Professorship in Medical Education, the requisite administrative staff, and the academic instructors at the individual hospitals and institutes who make up the interdisciplinary groups that will then collaboratively design each of the blocks. Furthermore, drafting specific regulations governing the medical school, the degree program and the theoretical and practical assessments is among the next tasks to be completed, as is the extensive construction on the hospital campus to build the needed space for teaching and research.

Setting up a new medical school at an existing university involves an extensive change process. Taking a targeted and strategic approach with regard for diverse external factors is critical to success. Not only must the existing structures be taken into account but also the many different broader influences (politics, economics, etc.) [26]. This new school will affect the Augsburg Hospital and those working there, the Augsburg University, the town of Augsburg and its environs, the other medical schools in Bavaria, and many other organizations, and even the people in the rest of Germany.

Most recently a new medical school was founded in 2012 at the University of Oldenburg and the European Medical School Oldenburg-Groningen (Fakultät für Medizin und Gesundheitswissenschaften). Relatively little is known about that process and the individual steps taken to establish the school or its effects on the people and places mentioned directly above. As a consequence, the change process here will be the subject of further study in addition to the founding of the Augsburg University Medical School, the refinement of the Augsburg curriculum, and the implementation of appropriate quality assurance measures to evaluate teaching and learning.

## Competing interests

The authors declare that they have no competing interests.
